# Early-Life Exposures and Early-Onset Uterine Leiomyomata in Black Women in the Sister Study

**DOI:** 10.1289/ehp.1103620

**Published:** 2011-11-02

**Authors:** Aimee A. D’Aloisio, Donna D. Baird, Lisa A. DeRoo, Dale P. Sandler

**Affiliations:** 1 Epidemiology Branch, National Institute of Environmental Health Sciences, National Institutes of Health, Department of Health and Human Services, Research Triangle Park, North Carolina, USA

**Keywords:** diabetes mellitus, diethylstilbestrol, early-life, leiomyoma, multiple birth offspring, pregnancy, pregnancy-induced hypertension, prenatal exposure delayed effects, socioeconomic factors, soy formula

## Abstract

Background: Uterine leiomyomata (fibroids) are hormonally responsive tumors, but little is known about risk factors. Early-life exposures may influence uterine development and subsequent response to hormones in adulthood. An earlier analysis of non-Hispanic white women who participated in the Sister Study found associations between several early-life factors and early-onset fibroids.

Objectives: We evaluated associations of early-life and childhood exposures with early-onset fibroids among black women and compared the results with those found among white women.

Methods: We analyzed baseline data from 3,534 black women, 35–59 years of age, in the Sister Study (a nationwide cohort of women who had a sister diagnosed with breast cancer) who self-reported information on early-life and childhood exposures. Early-onset fibroids were assessed based on self-report of a physician diagnosis of fibroids by the age of 30 years (*n* = 561). We estimated risk ratios (RR) and 95% confidence intervals (CI) from log-binomial regression models.

Results: Factors most strongly associated with early-onset fibroids were *in utero* diethylstilbestrol (DES; RR = 2.02; 95% CI: 1.28, 3.18), maternal prepregnancy diabetes or gestational diabetes (RR = 1.54; 95% CI: 0.95, 2.49), and monozygotic multiple birth (RR = 1.94; 95% CI: 1.26, 2.99). We also found positive associations with having been taller or thinner than peers at the age of 10 years and with early-life factors that included being the firstborn child of a teenage mother, maternal hypertensive disorder, preterm birth, and having been fed soy formula.

Conclusions: With the exception of monozygotic multiple birth and maternal hypertensive disorder, early-life risk factors for early-onset fibroids for black women were similar to those found for white women. However, in contrast to whites, childhood height and weight, but not low socioeconomic status indicators, were associated with early-onset fibroids in blacks. The general consistency of early-life findings for black and white women supports a possible role of early-life factors in fibroid development.

Uterine leiomyomata (fibroids) are highly prevalent benign tumors (Baird et al. 2003) that are the principal indication for hysterectomies in the United States (Farquhar and Steiner 2002). Fibroid-related morbidity may include pelvic pain, heavy bleeding, and reproductive problems (Stewart 2001). Black women have an increased risk of developing fibroids and greater severity of symptoms than do white women (Baird et al. 2003; Faerstein et al. 2001; Kjerulff et al. 1996; Marshall et al. 1997; Wise et al. 2005b). Although fibroids may be asymptomatic, prevalence estimates based on clinically evident disease have been as high as 50% for blacks and 25% for whites (Baird et al. 2003; Stewart 2001).

Although it is known that hormones influence fibroid development (Marsh and Bulun 2006), little is known about the risk factors that could explain these hormonal mechanisms. One hypothesis is that fibroid pathogenesis is influenced by early-life and childhood exposures that may affect the development of the uterus and later impact how a woman responds to hormonal challenges as an adult (Baird 2004). Proof of principle for this hypothesis is based on neonatal laboratory rodents exposed to experimental diethylstilbestrol (DES). In response to DES exposure, expression of estrogen-responsive genes was developmentally reprogrammed in the neonatal uterus, leading to fibroid development after maturity (Greathouse et al. 2008). Consistent with the laboratory animal findings, early data from non-Hispanic white participants in the Sister Study showed suggestive evidence of an elevated risk of early-onset uterine fibroids for women with *in utero* DES exposure (D’Aloisio et al. 2010). Other factors associated with early-onset fibroid development were having a mother diagnosed with diabetes before the index pregnancy (leading to participant’s birth), early gestational age at birth, having been fed soy formula, and low childhood socioeconomic status (D’Aloisio et al. 2010). At the time of our previous analysis, enrollment of the Sister Study cohort was ongoing, and we did not have sufficient numbers to evaluate associations with early-life factors for black women. With enrollment now complete, we were able to evaluate whether early-life and childhood exposures were associated with self-reported early-onset fibroids using baseline data from black participants in the Sister Study and to compare these results with associations found among non-Hispanic whites from the complete cohort. Our study examined early-life factors related to the mother’s index pregnancy and participant’s birth and feeding during infancy, as well as childhood developmental and socioeconomic factors.

## Materials and Methods

*Study population.* The Sister Study [National Institute of Environmental Health Sciences (NIEHS) 2011] is a prospective cohort study of 50,884 U.S. and Puerto Rican volunteer women 35–74 years of age who were not previously diagnosed with breast cancer but had a full or half sister who was diagnosed with breast cancer. Details of the study design have been described elsewhere (D’Aloisio et al. 2010). Study enrollment occurred between 2003 and 2009. The Sister Study was approved by the Institutional Review Board (IRB) of the NIEHS, National Institutes of Health, and the Copernicus Group IRB; all participants provided their informed consent. Computer-assisted telephone interviews assessed demographics, medical history, and possible risk factors for breast cancer and other conditions. Participants also completed a self-administered questionnaire on family history that included early-life events. Prepaid phone cards were given to participants to contact their mother or other relatives for assistance with completing family history questionnaires.

Our analysis included 3,534 black women who were 35–59 years of age when they completed baseline enrollment activities in the Sister Study. Similar to our previous analysis, we excluded women ≥ 60 years of age because of temporal trends in ultrasound use for fibroid diagnosis and because of the reduced likelihood that these women had a living mother to ask about early-life events (D’Aloisio et al. 2010). A total of 3,567 black women, 35–59 years of age, were available at baseline. We excluded 10 women with missing data on fibroid status, 21 with missing age at fibroid diagnosis, and 2 with unlikely ages at fibroid diagnosis—for example, fibroids are rarely seen in girls < 12 years of age who have not begun menstruation. For comparison to results in black women, we updated our previously published results in non-Hispanic white women (*n* = 19,972), which were from an analysis done prior to completion of cohort enrollment (D’Aloisio et al. 2010). Data from 27,786 non-Hispanic white women (hereafter referred to as “whites”) who were 35–59 years of age at baseline and who had complete data on fibroid diagnoses constituted the updated comparison group.

*Fibroid assessment.* During the baseline telephone interview, women reported whether a physician or health professional had ever diagnosed them as having uterine fibroids and their age at diagnosis. Because fibroids are highly prevalent and because their prevalence increases with age until menopause, many older women have undiagnosed fibroids. Therefore, we focused our study on early-onset fibroids to limit misclassification of women as noncases. For white women, we previously defined early-onset fibroids as a diagnosis at ≤ 35 years of age (D’Aloisio et al. 2010) based on ultrasound screening results that have shown that fibroid prevalence accelerates after 35 years of age (Baird et al. 2003; Laughlin et al. 2010). However, Laughlin et al. (2010) reported that the estimated age at onset may be about 10 years earlier for black women. Because our fibroid diagnoses are based on self-reported data, we used a conservative estimate to define early-onset fibroids in black women as a reported diagnosis at ≤ 30 years of age. We considered noncases to be all women who did not report early-onset fibroids, regardless of whether they reported a later fibroid diagnosis.

*Exposure and covariate assessment.* As previously described, most of the early-life exposures were ascertained in self-administered family history questionnaires that were completed at baseline (D’Aloisio et al. 2010). Early-life exposures included birth weight (pounds/ounces), categorical gestational age at birth, singleton or multiple birth, ever breastfed during infancy, ever fed soy formula, and maternal factors involving the index pregnancy (maternal age at birth, residence and work on a farm, smoking, DES use, prepregnancy diabetes, gestational diabetes, preeclampsia, and gestational hypertension). Gestational diabetes was defined as reporting gestational diabetes during the index pregnancy and reporting no history of diabetes before the index pregnancy. We defined mothers as having had gestational hypertension during the index pregnancy only if pregnancy-related high blood pressure was reported in the absence of preeclampsia. To allow for uncertainty, response options were “definitely,” “probably,” “probably not,” and “definitely not” as well as “don’t know” for most early-life questions. However, “don’t know” was the only option available to address uncertainty for gestational age, birth weight, singleton or multiple birth, and maternal age at birth.

Questionnaires provided additional information on multiple births including number of babies delivered from the index pregnancy, sex of multiple birth sibling(s), and zygosity based on the participant’s perception and test results when available. Using this information, we assigned twin births as monozygotic (reporting a twin sister who is genetically identical to the participant based on perception or testing), dizygotic (reporting a twin brother or a twin sister who is not genetically identical to the participant), or unknown (missing data to categorize type of twin birth). For the < 1% of black or white participants who reported having two or more multiple birth siblings, we combined them with twin birth categories according to the following criteria: monozygotic (all were genetically identical sisters), dizygotic (no sisters that are genetically identical to the participant), or unknown (missing data to categorize multiple births) or polyzygotic (having a genetically identical sister and a non–identical multiple birth sibling). Birth order was calculated using birthdates reported by participants for full siblings and half-siblings sharing the same mother. Participants reported sisters’ birthdates during the telephone interview and brothers’ birthdates in the family history questionnaire. Questionnaires also asked how long participants were breastfed or fed with soy formula, and whether soy formula consumption occurred during the first two months of life. Whether the participant’s mother was alive at baseline was reported in the family history questionnaire.

Participants reported childhood exposures during the baseline telephone interview. Childhood socioeconomic factors included educational level of parents or guardians in the household when the participant was 13 years of age, family income level based on self-reported categories (poor, low, middle, or well off), and food insecurity (not having enough to eat at times during childhood). Developmental factors were age at menarche and height and weight relative to peers at age 10 years. Information on participant characteristics at baseline, including age, education, smoking status, alcohol intake, ages at live and still births, and menopausal status, were assessed during the telephone interview. Body mass index at baseline was calculated using home visit measurements of weight (pounds) and height (inches).

*Statistical analyses*. We estimated risk ratios (RR) with 95% confidence intervals (CI) using log-binomial regression for associations between each exposure and early-onset fibroid diagnosis. For infancy feeding and maternal exposures involving the index pregnancy (except for maternal age at birth), women were classified as exposed if they reported “definitely” or “probably” for a given factor and as unexposed if they reported “probably not” or “definitely not.” We did not analyze data on how long participants were breast-fed or fed with soy formula because a substantial proportion of participants did not know this information. For birth weight analyses, we created the following categories: < 2,500 g (low), 2,500–3,999 g, and ≥ 4,000 g (high) based on clinical definitions of low and high birth weight and similarity of fibroid risk within categories. For maternal age and birth order, we created a combined index variable based on similarities in associations with fibroids and correlations between young maternal age at birth and being the firstborn using the following categories: < 20 years old and firstborn, ≥ 20 years old and firstborn, < 20 years old and not firstborn, and ≥ 20 years old and not firstborn.

We considered variables as confounders if they either influenced reporting or were associated with the fibroid outcome and early-life and childhood exposures. Specifically, in all race-specific log-binomial regression models, we included participant’s age and education because they may affect reporting of fibroids and exposures, and we included the maternal age at birth/birth-order index variable because it was associated with fibroids in our data and influences other early-life and childhood factors. We also adjusted for childhood family income in analyses for whites but not for blacks because childhood family income was only associated with the fibroid outcome for whites. Because of missing data on confounders, our adjusted analyses are based on 3,201 black women and 27,048 white women. For many of the observations excluded from the adjusted analyses, women did not complete family history questionnaires (*n* = 203 blacks; *n* = 361 whites), which resulted in missing data for maternal age at birth, birth order, and other early-life exposures. Despite evidence that early menarche is a risk factor for fibroids (Marshall et al. 1998; Wise et al. 2004), we did not adjust for age at menarche because it may be on the causal pathway between exposures of interest and fibroids. In secondary analyses, we further addressed whether other early-life factors may be acting as confounders by reevaluating associations after restricting analyses to smaller subsets of women without the potentially related factors. For example, we reevaluated the association with DES after excluding maternal conditions involving the index pregnancy (diabetes and hypertensive disorder). We conducted statistical analyses using SAS software (version 9.2; SAS Institute Inc., Cary, NC).

To assess whether our primary results for blacks were biased due to a high proportion of missing data for some early-life exposures, we also estimated associations with early-life exposures after performing multiple imputation analyses. These analyses assumed that missing data were not related to the actual values of missing variables but may have been associated with values of other study variables (i.e., we assumed that data were missing at random). We obtained ten imputation data sets from multiple imputation by chained equations using IVEware (version 0.1; University of Michigan, Ann Arbor, MI), which fits a series of regression models conditional on other variables and imputes missing data based on the values predicted for each participant in these models (Raghunathan et al. 2001, 2009). The multiple imputation regression models included all early-life factors (excluding soy formula consumption in the first two months of life because of multicollinearity with any soy formula consumption), childhood family income, participant’s age, participant’s education, mother’s vital status at baseline, and early-onset fibroid status. Childhood family income and maternal vital status were not confounders in log-binomial regression models; however, they were included in multiple imputation regression models because they influenced whether early-life data were missing. Log-binomial regression analyses for associations with early-onset fibroids were repeated in the 10 imputation data sets as previously described, and regression estimates were combined using PROC MIANALYZE in SAS (version 9.2; SAS Institute Inc.). In secondary analyses after imputation of missing data, we also explored whether there was possible confounding among the early-life exposures by adjusting for additional early-life factors in log-binomial regression models. Multiple imputation of missing data allowed for simultaneous adjustment for several early-life factors in log-binomial regression models without reductions in sample size.

## Results

The overall prevalence of self-reported fibroids among black women was 52%. Early-onset fibroids (diagnosed at ≤ 30 years of age) were reported by 16% of black women (Table 1). Black women who reported early-onset fibroids (cases) tended to be older at baseline (45–59 years of age, 82% vs. 79% of noncases) and were more likely to have received at least an associate’s or technical degree (74% vs. 67%) than were women who did not report an early-onset diagnosis (Table 1). In addition, proportionally more cases (33%) than noncases (29%) reported no live or still births before 31 years of age, and cases were much more likely than were noncases to have reported that menarche began at ≤ 11 years of age (33% vs. 24%) and that they were surgically menopausal at baseline (56% vs. 33%) (Table 1). Furthermore, cases were three times as likely to have had a hysterectomy at ≤ 35 years of age than were noncases (26% vs. 8%), which suggests that many of the cases experienced severe fibroid-related morbidity.

**Table 1:** Baseline characteristics [*n* (%)] by self-reported diagnosis of early-onset uterine fibroids among black women, 35–59 years of age, in the Sister Study, 2003–2009 (*n* = 3,534).*^a^*

Characteristic	Cases	Noncases
Total	561	2,973
Age (years)	
35-44	102 (18.2)	634 (21.3)
45-59	459 (81.8)	2,339 (78.7)
Highest level of education	
High school or less^*b*^	32 (5.7)	310 (10.4)
Some college, no degree	116 (20.7)	659 (22.2)
Associate's or technical degree	104 (18.5)	391 (13.2)
Bachelor's degree	167 (29.8)	859 (28.9)
Graduate degree	142 (25.3)	753 (25.3)
Missing	0	1
Maternal living status	
Alive	276 (52.9)	1,498 (53.6)
Deceased	246 (47.1)	1,296 (46.4)
Missing^*c*^	39	179
Smoking status	
Current	76 (13.5)	352 (11.8)
Former	131 (23.4)	688 (23.1)
Never	354 (63.1)	1,933 (65.0)
Alcohol intake (drinks/week)	
0	143 (25.6)	847 (28.6)
0.01-0.49	231 (41.3)	1,124 (37.9)
0.50-2.00	104 (18.6)	511 (17.3)
2.01-6.99	55 (9.8)	314 (10.6)
≥ 7.00	26 (4.7)	166 (5.6)
Missing	2	11
Body mass index	
< 25	77 (13.8)	514 (17.6)
25-29.9	173 (31.0)	905 (30.9)
≥ 30	308 (55.2)	1,508 (51.5)
Missing	3	46
Age at menarche (years)	
< 11	88 (15.7)	292 (9.8)
11	95 (16.9)	419 (14.1)
12-13	278 (49.6)	1,574 (53.0)
14	58 (10.3)	308 (10.4)
≥ 15	42 (7.5)	376 (12.7)
Missing	0	4
Parity at age 30 years^d^	
0	184 (32.9)	865 (29.2)
1	178 (31.8)	837 (28.3)
2	134 (24.0)	823 (27.8)
≥ 3	63 (11.3)	437 (14.8)
Missing	2	11
Menopausal status^*e*^	
Premonopausal	159 (28.3)	1,276 (43.0)
Natural	87 (15.5)	727 (24.5)
Surgical	315 (56.1)	965 (32.5)
Missing	0	5
**a**Cases are women with self-reported diagnosis of fibroids at ≤ 30 years of age. **b**Less than high school includes 3 cases and 35 noncases. **c**Data were missing for 203 women because they did not complete family history questionnaires. **d**Total number of live and still births before 31 years of age. **e**Natural menopause includes women who had not had a menstrual period in the past 12 months as long as they were not pregnant, breastfeeding, or using premenopausal medications that cause amenorrhea. Surgical menopause includes women whose menstrual periods had ended before natural menopause because they had had a hysterectomy, bilateral oophorectomy, endometrial ablation, uterine artery embolization, or chemotherapy.

*Early-life factors.* Eight early-life factors (DES, diabetes, hypertensive disorder, being the firstborn child of a teenage mother, low birth weight, early gestational age at birth, multiple birth, and soy formula) were associated with at least a 20% relative increase in the risk of early-onset fibroid diagnosis among black women (Table 2). The strongest associations were with *in utero* DES (RR = 2.02; 95% CI: 1.28, 3.18) and maternal prepregnancy diabetes or gestational diabetes (RR = 1.54; 95% CI: 0.95, 2.49). The association with gestational diabetes (RR = 2.09; 95% CI: 1.00, 4.34) was stronger than the association with prepregnancy diabetes (Table 2). Excluding women who reported that their mothers had medical conditions during the index pregnancy (hypertensive disorder and prepregnancy diabetes or gestational diabetes) did not affect the association with *in utero* DES (RR = 1.95; 95% CI: 1.15, 3.30) based on 2,240 observations. In our previously published study of whites (D’Aloisio et al. 2010), we found that the association with DES was driven by

**Table 2:** RRs for early-onset uterine fibroids*a* in association with early-life exposures among black women, 35–59 years of age, in the Sister Study, 2003–2009.

Exposure	Total (*n*=3,201)^b^	Cases (*n*)	Adjusted RR (95% CI)
Maternal pregnancy factors	
Farm^*c*^	
Work and residence	418	59	0.88 (0.68, 1.13)
Work only	83	13	0.99 (0.60, 1.64)
Residence only	85	22	1.48 (1.03, 2.15)
None	2,429	384	1.00
Missing	186	27
Smoking status^*c*^	
Definitely/probably	738	130	1.18 (0.98, 1.42)
Definitely not/probably not	2,161	322	1.00
Missing	302	53
DES use^*c*^	
Definitely/probably	42	13	2.02 (1.28, 3.18)
Definitely not/probably not	2,532	368	1.00
Missing	807	124
Diabetes^*c*^	
Any	54	13	1.54 (0.95, 2.49)
Prepregnancy	36	8	1.38 (0.75, 2.55)
Gestational	16	5	2.09 (1.00, 4.34)
Unknown	2	0
None	2,598	407	1.00
Missing	549	85
Hypertensive disorder^*c*^	
Any	176	36	1.38 (1.01, 1.87)
Preeclampsia	62	15	1.38 (1.01, 1.87)
Gestational hypertension	82	17	1.41 (0.91, 2.17)
Unknown	32	4
None	2,046	310	1.00
Missing	979	159
Birth and infancy factors	
Maternal age at birth/birth order	
< 20 years/1st	183	41	1.43 (1.08, 1.90)
≥ 20 years/1st	261	34	0.83 (0.60, 1.15)
< 20 years/2nd or later	144	21	0.97 (0.65, 1.45)
≥ 20 years/2nd or later	2,613	409	1.00
Birth weight (g)	
< 2,500	335	67	1.31 (1.03, 1.66)
2,500-3,999	1,530	239	1.00
≥ 4,000	131	17	0.84 (0.53, 1.33)
Missing	1,205	182
Gestational age at birth	
Born ≥ 1 months early	83	19	1.43 (0.94, 2.16)
Born 2-4 weeks early	61	12	1.35 (0.79, 2.31)
Not born ≥ 2 weeks early	951	155	1.00
Missing	2,106	319
Multiple birth	
Any	133	29	1.42 (1.02, 1.97)
Monozygotic	43	14	1.94 (1.26, 2.99)
Dizygotic	69	10	0.98 (0.55, 1.75)
Unknown or polyzygotic	21	5
None	3,057	472	1.00
Missing	11	4
Fed breast milk^*c*^	
Definitely/probably	1,438	250	1.18 (0.99, 1.40)
Definitely not/probably not	1,385	200	1.00
Missing	378	55
Fed soy formula^*c*^	
Definitely/probably	96	19	1.26 (0.83, 1.89)
Definitely not/probably not	2,486	411	1.00
Missing	619	75
Fed soy formula (age ≤ 2 months)	
Yes	39	9	1.48 (0.84, 2.63)
No	2,553	422	1.00
Missing	609	74
**a**Early-onset uterine fibroid status was based on self-reported diagnosis at ≤ 30 years of age (adjusted: *n* = 505). **b**Each adjusted model included the following core variables: participant’s age and education and maternal age/birth-order index. Women with missing data for core variables were excluded from all models. **c”**Definitely” and “probably” were combined as affirmative responses, and “probably not” and “definitely not” were combined as negative responses.							

women who reported probable rather than definite exposure. However, we estimated associations separately for this analysis of black women who reported definite and probable exposure to *in utero* DES, and we found that RRs were comparable (definite, RR = 1.87; 95% CI: 1.00, 3.50; probable, RR = 2.22; 95% CI: 1.15, 4.26). The multiple birth association appeared to be specific to monozygotic multiple births (RR = 1.94; 95% CI: 1.26, 2.99) (Table 2). After restricting analyses to singleton births and excluding women born at least 1 month early, we observed little change in the association between low birth weight and early-onset fibroids (RR = 1.27; 95% CI: 0.94, 1.71 based on 1,833 observations). Although maternal farm residence during the index pregnancy was associated with early-onset fibroids, we found this association only among women who reported that their mothers had not worked on a farm during the index pregnancy.

*Childhood factors.* Two factors—having been taller and having been thinner at age 10 years compared with peers—were associated with ≥ 20% increases in the RRs of early-onset fibroids among blacks (Table 3). Childhood socioeconomic factors were not associated with ≥ 20% increases in the RRs of early-onset fibroids.

**Table 3:** RRs for early-onset uterine fibroids*^a^* in association with childhood socioeconomic and developmental factors among blacks, 35–59 years of age, in the Sister Study, 2003–2009.

Exposure	Total (*n* = 3,201)*b*	Cases (*n*)	Adjusted RR (95% CI)	
Maximum household education (participant age 13 years)							
< High school		1,050		160		0.94	(0.79, 1.12)
≥ High school		2,089		335		1.00	
Missing		62		10			
Family income							
Poor		557		90		1.02	(0.82, 1.28)
Low		1,134		181		1.01	(0.84, 1.21)
Middle/well off		1,499		232		1.00	
Missing		11		2			
Not enough to eat							
Yes		572		101		1.15	(0.94, 1.40)
No		2,623		404		1.00	
Missing		6		0			
Height relative to peers (age 10 years)							
Taller		832		154		1.24	(1.03, 1.50)
Same		1,419		212		1.00	
Shorter		942		139		0.99	(0.82, 1.21)
Missing		8		0			
Weight relative to peers (age 10 years)							
Heavier		538		83		1.09	(0.86, 1.38)
Same		1,343		190		1.00	
Thinner		1,304		231		1.23	(1.03, 1.46)
Missing		16		1			
**a**Early-onset uterine fibroid status was based on self-reported diagnosis at ≤ 30 years of age (adjusted: *n* = 505). **b**Each adjusted model included the following core variables: participant’s age and education and maternal age/birth-order index. Women with missing data for core variables were excluded from all models.							

**Figure 1:**
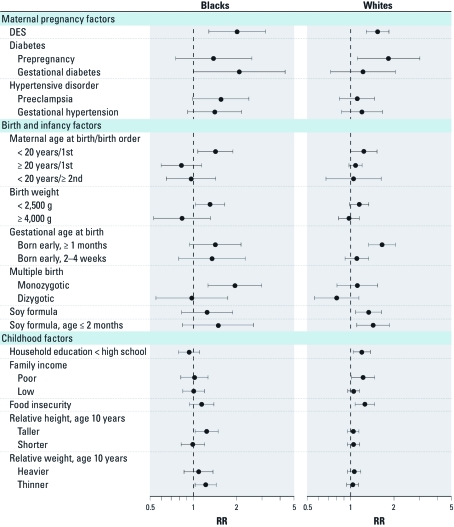
Comparisons in associations [RR (95% CI)] of selected early-life and childhood exposures with early-onset fibroids between black and white women, 35–59 years of age, in the Sister Study, 2003–2009.

*Comparisons between blacks and whites.* Early-life and childhood exposures that were associated with at least a 20% relative increase or decrease in early-onset fibroid risk for either black or white women are shown in Figure 1; we included results that we updated from the previous study of white women (D’Aloisio et al. 2010) [see Supplemental Material, Tables 1 and 2 (http://dx.doi.org/10.1289/ehp.1103620) for the estimated associations for all study exposures among the full cohort of eligible white women in the Sister Study]. The proportion of women who reported early-onset fibroids was 8% for white women (diagnosed at ≤ 35 years of age) versus 16% for black women (diagnosed at ≤ 30 years of age). Early-life factors associated with early-onset fibroids among blacks were similar to those among white women with the exception of monozygotic multiple birth status, which was only associated with early-onset fibroids among black women. In addition, associations with maternal hypertensive disorder and low birth weight were stronger among black women than among white women. For maternal diabetes, gestational diabetes was more strongly associated with early-onset fibroids than was prepregnancy diabetes among blacks, whereas the association was stronger for prepregnancy diabetes than gestational diabetes among whites (RR = 1.83; 95% CI: 1.10, 3.03 and RR = 1.22; 95% CI: 0.72, 2.05, respectively).

There were larger differences in associations with childhood exposures between black and white women. Taller height and thinner weight at age 10 years were associated with early-onset fibroids only for black women while associations with factors indicating low childhood socioeconomic status were generally evident only for whites.

*Imputation analyses.* Data on each of the early-life factors except maternal age at birth/birth-order index and multiple birth status were missing for at least 10% of black women. We performed multiple imputations of missing data for nearly all early-life factors and repeated our analyses of associations with fibroids [see Supplemental Material, Table 3 (http://dx.doi.org/10.1289/ehp.1103620)]. Results from multiple imputation analyses were generally consistent with those shown in Table 2. After imputation of missing data, we adjusted our analyses for additional early-life factors in log-binomial regression models to evaluate possible confounding. Associations with maternal diabetes and hypertensive disorder were unaffected by further adjustment for multiple birth status (results not shown). The *in utero* DES association was only slightly attenuated (RR = 1.68; 95% CI: 0.98, 2.87) after repeating analyses with adjustment for maternal conditions (diabetes and hypertensive disorder) and multiple birth status, which are factors associated with higher risk pregnancy and hence a potentially increased likelihood that mothers were prescribed DES. However, the association was weakened for low birth weight (RR = 1.12; 95% CI: 0.85, 1.47) but was similar for having been born at least 1 month early (RR = 1.35; 95% CI: 0.87, 2.08) after inclusion of both early-life factors in the same model and adjustment for *in utero* DES, maternal diabetes, maternal hypertensive disorder, and multiple birth status.

## Discussion

We found that black women had an increased risk of early-onset fibroids in association with several early-life factors. Many of these associations were also found among white women with the exception of maternal hypertensive disorder and monozygotic multiple birth status. For black women, we also found that early-onset fibroids were associated with taller height and thinner weight at 10 years of age compared with peers, but no associations were observed with indicators of low socioeconomic status during childhood.

Misclassification of self-reported fibroids is a potential bias because fibroid risk increases with age, and many women who develop fibroids may never be diagnosed by a physician (Laughlin et al. 2010). Therefore, we only considered early-onset diagnoses to improve sensitivity by reducing misclassification in the noncase group. For whites, we had defined early-onset cases as those occurring at ≤ 35 years of age (D’Aloisio et al. 2010); however, fibroids develop at an earlier age in blacks (Laughlin et al. 2010). Therefore, to reduce misclassification in the noncase group to the same degree in black women, we defined early-onset cases as those occurring at ≤ 30 years of age. We found that the estimated proportion of black women with early-onset fibroids in our study (16%) was consistent with the proportion of middle-aged black women (19%) with self-reported fibroids by age 30 years in the NIEHS Uterine Fibroid Study (Baird DD, unpublished data). In addition, cases were much more likely than noncases to report surgical menopause and early hysterectomies, which are expected consequences for women with fibroids, and this supports that the specificity of self-reported early-onset fibroids is likely to be very high in our study.

Given that the majority of exposures occurred *in utero* or during infancy, exposure misclassification is another potential source of bias. However, participants received prepaid phone cards to encourage them to contact their mothers or other relatives to ask about early-life events, and we excluded women > 59 years of age who were less likely to have living relatives to consult. In addition, response categories for most of the early-life exposures allowed for additional uncertainty in reporting with definite and probable categories. We repeated the analyses by evaluating associations separately for the reporting of definite and probable exposure, and we did not find that the reported associations with DES or any of the other early-life factors were unduly influenced by reporting probable exposure (results not shown). It is unlikely that differential exposure misclassification as a result of recall bias would have occurred in our study because none of the exposures are established risk factors for fibroids. Instead, any exposure misclassification in our study would likely be nondifferential, which would generally result in RR estimates biased toward the null.

Strengths of this study include a focus on black women who have been shown to have greater fibroid incidence and morbidity than do white women (Baird et al. 2003; Faerstein et al. 2001; Kjerulff et al. 1996; Marshall et al. 1997; Wise et al. 2005b). Despite the small numbers of exposed cases for many of the early-life exposures, we were able to replicate many of the early-life findings from our previous analysis of white women with the exception of unique associations with monozygotic multiple birth status and maternal hypertensive disorder in black women. However, we found larger differences between blacks and whites in associations with childhood factors (D’Aloisio et al. 2010). Although > 10% of black women had missing data for most of the early-life exposures, results of multiple imputation analyses suggest that missing data did not bias reported associations.

*In utero* DES was one of the early-life exposures that was most strongly associated with early-onset fibroids among black women. Furthermore, the association between *in utero* DES and early-onset fibroids remained after excluding women who reported that mothers had medical conditions (diabetes and hypertensive disorder) during the index pregnancy, which may have increased the likelihood of treatment with DES. Therefore, these conditions were unlikely to have confounded the association with DES. Evidence from animal studies shows that DES exposure during early development can increase fibroid incidence in adulthood (Cook et al. 2007). Previous epidemiological studies of *in utero* DES and fibroids have reported inconsistent findings, possibly due to differences in study design and assessment of DES exposure and fibroids (Baird and Newbold 2005; Missmer et al. 2004; Wise et al. 2005a). In particular, Wise et al. (2005a) found no association between medically documented DES exposure and surgical fibroid cases in a prospective study of women from two cohorts. Using data from a prospective study of endometriosis in Nurses’ Health Study participants with intact uteri, Missmer et al. (2004) noted that there was no association between self-reported DES exposure and uterine fibroids. However, Baird and Newbold (2005) indicated a positive association with self-reported DES exposure based on small numbers of exposed cases among black and white women from the NIEHS Uterine Fibroid Study, which used ultrasound screening to identify fibroids and included black women in about 60% of the study sample. In addition, our finding for black women was similar to that for white women in the Sister Study. However, it is possible that women in the Sister Study who reported DES exposure may have received more gynecologic screenings, which could have led to a greater likelihood of detecting early-onset fibroids. Further studies of DES are needed in different study populations to support involvement in fibroid pathogenesis.

Another *in utero* factor associated with fibroids in black women was maternal diabetes—this association was slightly stronger for gestational diabetes than for prepregnancy diabetes. However, associations for gestational and prepregnancy diabetes were more similar in analyses after multiple imputation of missing data. Among whites, we also found a similar association with maternal diabetes, although the association was strongest for prepregnancy diabetes. We did not have information on the status of post-pregnancy diabetes in the participants’ mothers, and distinguishing gestational diabetes from young onset of type 2 diabetes is difficult because limited access to care may have influenced whether mothers received a diagnosis of diabetes before pregnancy. If black women had poorer access to care than did white women, this may explain the differences in results for the two groups. As previously discussed, biological support exists for *in utero* exposure to diabetes influencing later risk of fibroids via developmental changes that may affect epigenetic patterns in areas pertaining to regulation of relevant genes for fibroid pathogenesis (D’Aloisio et al. 2010). There were few cases of maternal diabetes among black women in the cohort possibly reflecting limitations in available medical care for participants’ mothers. In the United States, blacks have greater rates of type 2 and gestational diabetes than do whites (Egede and Dagogo-Jack 2005; Savitz et al. 2008); therefore, blacks are more likely to have *in utero* exposure to hyperinsulinemia and hyperglycemia that result in adverse developmental consequences. Thus, *in utero* exposure to maternal diabetes may contribute to the elevated fibroid burden among U.S. black women.

We previously reported an association between soy formula and early-onset fibroids among whites (D’Aloisio et al. 2010). Associations with having been fed soy formula (ever fed soy formula and fed soy formula within the first 2 months of life) were of similar magnitude for blacks and for whites, although the estimates for blacks were more imprecise. As previously discussed, soy formula might influence later risk of fibroids because of the high concentration of estrogenic isoflavones (D’Aloisio et al. 2010). This hypothesis is further supported by effects on the reproductive system observed in neonatal laboratory rodents administered genistein, the hormonally activated form of the isoflavone predominantly found in soy formula (Jefferson and Williams 2011). There have been few studies of soy formula in relation to reproductive consequences in humans. However, one study reported that having been fed soy formula was associated with longer menstrual bleeding and greater pain with menstruation (Strom et al. 2001), both of which are also symptoms of fibroids.

Our study of black women is the first to report a strong association between early-onset fibroid risk and multiple birth, specifically for having been part of a monozygotic multiple birth. A possible causal mechanism is unclear but may be due to a greater likelihood of fetal growth restriction and resulting metabolic consequences in multiple births versus singleton births. More than half of monozygotic multiple births are monochorionic, which may lead to a greater risk of adverse outcomes than for dizygotic multiple births (Vaag and Poulsen 2007). We also found an association with maternal hypertensive disorder that persisted after accounting for multiple birth status. Preeclampsia and gestational hypertension are more likely to occur in the U.S. black population than in whites (Tanaka et al. 2007). Maternal hypertensive disorder may affect placental development leading to fetal malnourishment, which has been associated with increased risk of metabolic diseases in adulthood (Anderson 2007). Among black women, there were associations with early gestational age at birth and with low birth weight, which remained after excluding multiple births and those born at least one month before their due date. However, approximately 40% of women were missing birth weight data, and > 60% were missing data on gestational age at birth; adjusting for other early-life confounders in multiple imputation analyses resulted in attenuation of the association with low birth weight, although an association remained with having been born at least 1 month early. An association with having been born preterm was also found among white women, but the RR estimate for whites was more precise than for blacks. We previously discussed the possible biological plausibility for an association with having been born preterm based on not receiving enough estrogen for adequate uterine development (D’Aloisio et al. 2010).

With the possible exception of having a teenage mother, we did not find associations between fibroids and indicators of low socioeconomic status during childhood among black women, as we did for whites. Low childhood socioeconomic status indicators such as parent education and self-reported family income level may have a different societal context for blacks and whites, which may differentially influence adult health outcomes (Shavers 2007). Nevertheless, it is also possible that other sources of stress such as perceived racism (Wise et al. 2007) may have more relevance in fibroid pathogenesis for black women.

We also found associations with self-reported indicators of childhood height and weight among blacks but not among whites. Specifically, we found early-onset fibroid associations with having been taller than peers at age 10 years and having been thinner than peers at age 10 years. These associations did not change after including both these variables in the same model (results not shown), which suggests that these are independent associations. Even though early menarche is a risk factor for fibroids (Marshall et al. 1998; Wise et al. 2004), it may be on the causal pathway because of its relations with greater childhood body fat (Ahmed et al. 2009) and taller childhood height (Freedman et al. 2002). Nevertheless, we verified that further adjustment for age at menarche did not affect our findings with childhood weight and height (results not shown). We also further excluded women who reported low birth weight (because size at birth may influence later childhood weight) and found that the association with thinner childhood weight did not change (result not shown). However, we acknowledge that self-reporting by adults of childhood height and weight relative to peers has limitations in that they are not absolute measures of height or weight and reflect participants’ perceptions of their size relative to peers as well as their peer group. Terry et al. (2007) reported no association with childhood body fatness using data from the Nurses’ Health Study, which is consistent with our findings in whites.

## Conclusions

Our findings in blacks replicate many of our previously published associations with early-life factors and fibroids in whites (D’Aloisio et al. 2010). Biologically plausible mechanisms exist for these factors to promote fibroid pathogenesis in adulthood. Despite largely consistent findings between blacks and whites, most of these early-life factors have not been investigated in other study populations, so further replication is needed to support their possible involvement in fibroid pathogenesis. Although many exposures such as diabetes and pregnancy complications were relatively rare in our cohort, their prevalence in the general population is likely growing given the obesity epidemic in the United States.

## Supplemental Material

(78 KB) PDF

## References

[r1] Ahmed ML, Ong KK, Dunger DB (2009). Childhood obesity and the timing of puberty.. Trends Endocrinol Metab.

[r2] Anderson CM (2007). Preeclampsia: exposing future cardiovascular risk in mothers and their children.. J Obstet Gynecol Neonatal Nurs.

[r3] Baird DD (2004). Invited commentary: uterine leiomyomata—we know so little but could learn so much.. Am J Epidemiol.

[r4] Baird DD, Dunson DB, Hill MC, Cousins D, Schectman JM (2003). High cumulative incidence of uterine leiomyoma in black and white women: ultrasound evidence.. Am J Obstet Gynecol.

[r5] Baird DD, Newbold R (2005). Prenatal diethylstilbestrol (DES) exposure is associated with uterine leiomyoma development.. Reprod Toxicol.

[r6] Cook JD, Davis BJ, Goewey JA, Berry TD, Walker CL (2007). Identification of a sensitive period for developmental programming that increases risk for uterine leiomyoma in Eker rats.. Reprod Sci.

[r7] D’Aloisio AA, Baird DD, DeRoo LA, Sandler DP (2010). Association of intrauterine and early-life exposures with diagnosis of uterine leiomyomata by 35 years of age in the Sister Study.. Environ Health Perspect.

[r8] Egede LE, Dagogo-Jack S (2005). Epidemiology of type 2 diabetes: focus on ethnic minorities.. Med Clin North Am.

[r9] Faerstein E, Szklo M, Rosenshein N. (2001). Risk factors for uterine leiomyoma: a practice-based case-control study. I. African-American heritage, reproductive history, body size, and smoking.. Am J Epidemiol.

[r10] Farquhar CM, Steiner CA (2002). Hysterectomy rates in the United States 1990-1997.. Obstet Gynecol.

[r11] Freedman DS Khan LK Serdula MK Dietz WH Srinivasan SR Berenson GS 2002 Relation of age at menarche to race, time period, and anthropometric dimensions: the Bogalusa Heart Study. Pediatrics 110 4 e43; doi:10.1542/peds.110.4.e43 [Online 13 June 2002] 12359816

[r12] Greathouse KL, Cook JD, Lin K, Davis BJ, Berry TD, Bredfeldt TG (2008). Identification of uterine leiomyoma genes developmentally reprogrammed by neonatal exposure to diethylstilbestrol.. Reprod Sci.

[r13] Jefferson WN, Williams CJ (2011). Circulating levels of genistein in the neonate, apart from dose and route, predict future adverse female reproductive outcomes.. Reprod Toxicol.

[r14] Kjerulff KH, Langenberg P, Seidman JD, Stolley PD, Guzinski GM (1996). Uterine leiomyomas. Racial differences in severity, symptoms and age at diagnosis.. J Reprod Med.

[r15] Laughlin SK, Schroeder JC, Baird DD (2010). New directions in the epidemiology of uterine fibroids.. Semin Reprod Med.

[r16] Marsh EE, Bulun SE (2006). Steroid hormones and leiomyomas.. Obstet Gynecol Clin North Am.

[r17] Marshall LM, Spiegelman D, Barbieri RL, Goldman MB, Manson JE, Colditz GA (1997). Variation in the incidence of uterine leiomyoma among premenopausal women by age and race.. Obstet Gynecol.

[r18] Marshall LM, Spiegelman D, Goldman MB, Manson JE, Colditz GA, Barbieri RL (1998). A prospective study of reproductive factors and oral contraceptive use in relation to the risk of uterine leiomyomata.. Fertil Steril.

[r19] Missmer SA, Hankinson SE, Spiegelman D, Barbieri RL, Michels KB, Hunter DJ (2004). *In utero* exposures and the incidence of endometriosis.. Fertil Steril.

[r20] NIEHS (National Institute of Environmental Health Sciences) (2011). The Sister Study.. http://www.sisterstudy.org/.

[r21] Raghunathan TE, Lepkowski JM, Van Hoewyk J, Solenberger P (2001). A multivariate technique for multiply imputing missing values using a sequence of regression models.. Surv Methodol.

[r22] Raghunathan TE, Solenberger PW, Van Hoewyk J (2009). IVEware: Imputation and Variance Estimation Software. Ann Arbor, MI: Survey Methodology Program, Survey Research Center, Institute for Social Research, University of Michigan.. http://www.isr.umich.edu/src/smp/ive/.

[r23] Savitz DA, Janevic TM, Engel SM, Kaufman JS, Herring AH (2008). Ethnicity and gestational diabetes in New York City, 1995-2003.. BJOG.

[r24] Shavers VL (2007). Measurement of socioeconomic status in health disparities research.. J Natl Med Assoc.

[r25] Stewart EA (2001). Uterine fibroids.. Lancet.

[r26] Strom BL, Schinnar R, Ziegler EE, Barnhart KT, Sammel MD, Macones GA (2001). Exposure to soy-based formula in infancy and endocrinological and reproductive outcomes in young adulthood.. JAMA.

[r27] Tanaka M, Jaamaa G, Kaiser M, Hills E, Soim A, Zhu M (2007). Racial disparity in hypertensive disorders of pregnancy in New York State: a 10-year longitudinal population-based study.. Am J Public Health.

[r28] Terry KL, De Vivo I, Hankinson SE, Spiegelman D, Wise LA, Missmer SA (2007). Anthropometric characteristics and risk of uterine leiomyoma.. Epidemiology.

[r29] Vaag A, Poulsen P. (2007). Twins in metabolic and diabetes research: what do they tell us?. Curr Opin Clin Nutr Metab Care.

[r30] Wise LA, Palmer JR, Cozier YC, Hunt MO, Stewart EA, Rosenberg L (2007). Perceived racial discrimination and risk of uterine leiomyomata.. Epidemiology.

[r31] Wise LA, Palmer JR, Harlow BL, Spiegelman D, Stewart EA, Adams-Campbell LL (2004). Reproductive factors, hormonal contraception, and risk of uterine leiomyomata in African-American women: a prospective study.. Am J Epidemiol.

[r32] Wise LA, Palmer JR, Rowlings K, Kaufman RH, Herbst AL, Noller KL (2005a). Risk of benign gynecologic tumors in relation to prenatal diethylstilbestrol exposure.. Obstet Gynecol.

[r33] Wise LA, Palmer JR, Stewart EA, Rosenberg L (2005b). Age-specific incidence rates for self-reported uterine leiomyomata in the Black Women’s Health Study.. Obstet Gynecol.

