# Supplementary material for: Early-Life Exposures and Early-Onset Uterine Leiomyomata in Black Women in the Sister Study
Source: Environ Health Perspect. 2011 Nov 2;120(3):406–12. doi: 10.1289/ehp.1103620 (PMC3295338; doi:10.1289/ehp.1103620)
Supplement: (78 KB) PDF [file ehp.1103620.s001.pdf]

Supplemental Material

Early-Life Exposures and Early-Onset Uterine Leiomyomata in Black Women in the Sister Study

Aimee A. D'Aloisio, Donna D. Baird, Lisa A. DeRoo, Dale P. Sandler

**Outline**	**Pages**
Supplemental Material, Table 1	2-3
Supplemental Material, Table 2	4
Supplemental Material, Table 3	5-6

Supplemental Material, Table 1. Risk ratios for early onset uterine fibroids in association with early-life exposures in whites, 35-59 years, in the complete Sister Study cohort, 2003-2009[a]

Exposure	Total (n = 27,048)[b]	Cases (n)	Adjusted RR	95% CI
Maternal Pregnancy Factors:				
Farm[c]				
Work and residence	2,267	180	0.99	0.85, 1.16
Work only	298	24	1.04	0.71, 1.53
Residence only	1,033	73	0.89	0.71, 1.12
No	22,928	1,739	1.00	
Missing	522	49		
Smoking[c]				
Definitely/probably	9,484	735	1.03	0.94, 1.13
Definitely not/probably not	16,682	1,246	1.00	
Missing	882	84		
DES use[c]				
Definitely/probably	893	102	1.53	1.27, 1.85
Definitely not/probably not	22,641	1,685	1.00	
Missing	3,514	278		
Diabetes[c]				
Any	245	26	1.45	1.00, 2.08
Pre-pregnancy	93	13	1.83	1.10, 3.03
Gestational	149	13	1.22	0.72, 2.05
Unknown	3	0		
None	24,838	1,878	1.00	
Missing	1,965	161		
Hypertensive disorder[c]				
Any	957	81	1.11	0.89, 1.37
Pre-eclampsia	526	45	1.10	0.83, 1.47
Gestational hypertension	359	32	1.19	0.86, 1.67
Unknown	72	4		
None	21,216	1,595	1.00	
Missing	4,875	389		
Participant Factors:				
Maternal age at birth/birth order				
<20 years, 1st	794	76	1.23	0.99, 1.53
≥20 years, 1st	4,389	358	1.09	0.98, 1.22
<20 years, 2nd or later	219	18	1.05	0.67, 1.64
≥20 years, 2nd or later	21,646	1,613	1.00	
Birth weight, grams				
<2,500	1,893	171	1.16	0.99, 1.35
2,500-3,999	17,971	1,376	1.00	
≥4,000	1,895	141	0.98	0.83, 1.15
Missing	5,289	377		

Supplemental Material, Table 1 (cont.)

Exposure	Total (n = 27,048)[b]	Cases (n)	Adjusted RR	95% CI
Gestational age at birth				
Born early, ≥1 months	621	75	1.65	1.32, 2.05
Born early, 2-4 weeks	1,379	110	1.11	0.92, 1.34
Not ≥2 weeks early	13,727	991	1.00	
Missing	11,321	889		
Multiple birth				
Any	924	64	0.90	0.71, 1.15
Monozygotic	382	33	1.12	0.80, 1.55
Dizygotic	474	29	0.80	0.56, 1.15
Unknown or polyzygotic	68	2		
None	26,077	1,996	1.00	
Missing	47	5		
Fed breast milk[c]				
Definitely/probably	9,805	717	0.92	0.84, 1.01
Definitely not/probably not	15,834	1,237	1.00	
Missing	1,409	111		
Fed soy formula[c]				
Definitely/probably	857	84	1.33	1.08, 1.64
Definitely not/probably not	22,061	1,654	1.00	
Missing	4,130	327		
Fed soy formula, age ≤2 months				
Yes	492	51	1.43	1.10, 1.86
No	22,349	1,677	1.00	
Missing	4,207	337		

Abbreviations: risk ratio, RR; CI, confidence interval; DES, diethylstilbestrol.

[a] Early onset uterine fibroid status was based on self-reported diagnosis at ≤ 35 years of age (adjusted: n = 2,065).

[b] Each adjusted model included the following core variables: participant's age and education, maternal age/birth order index, and childhood family income. Women with missing data for core variables were excluded from all models.

[c] Definitely and probably were combined as affirmative responses, and probably not and definitely not were combined as negative responses.

Supplemental Material, Table 2. Risk ratios early onset uterine fibroids in association with childhood socioeconomic and developmental factors in whites, 35-59 years, in the complete Sister Study cohort, 2003-2009[a]

Exposure	Total (n = 27,048)[b]	Cases (n)	Adjusted RR	95% CI
Maximum household education, participant age 13				
< High school	2,592	245	1.20	1.05, 1.37
≥ High school	24,330	1,809	1.00	
Missing	126	11		
Family income				
Poor	1,169	110	1.22	1.01, 1.47
Low	5,843	466	1.05	0.95, 1.16
Middle/well off	20,036	1,489	1.00	
Not enough to eat				
Yes	1,994	199	1.26	1.08, 1.47
No	25,048	1,865	1.00	
Missing	6	1		
Height relative to peers, age 10				
Taller	7,978	619	1.05	0.95, 1.16
Same	12,252	915	1.00	
Shorter	6,793	530	1.05	0.95, 1.16
Missing	25	1		
Weight relative to peers, age 10				
Heavier	5,458	429	1.06	0.95, 1.18
Same	12,505	931	1.00	
Lighter	9,047	701	1.04	0.94, 1.14
Missing	38	4		

Abbreviations: risk ratio, RR; CI, confidence interval.

[a] Early onset uterine fibroid status was based on self-reported diagnosis at ≤ 35 years of age (adjusted: n = 2,065).

[b] Each adjusted model included the following core variables: participant's age and education, maternal age/birth order index, and childhood family income. Women with missing data for core variables were excluded from all models.

Supplemental Material, Table 3. Multiple imputation of missing data for early-life exposures in association with early onset fibroids in blacks, 35-59 years, in the Sister Study, 2003-2009[a]

Exposure	Imputed %	Imputed (n = 3,534)[b] RR	95% CI
Maternal Pregnancy Factors:			
Farm[c]	11		
Work and residence		0.91	0.71, 1.16
Work only		0.93	0.55, 1.56
Residence only		1.51	1.05, 2.16
None		1.00	
Smoking[c]	15		
Definitely/probably		1.14	0.95, 1.37
Definitely not/probably not		1.00	
DES use[c]	30		
Definitely/probably		1.75	1.11, 2.74
Definitely not/probably not		1.00	
Diabetes[c]	23		
Any		1.39	0.83, 2.30
Pre-pregnancy		1.35	0.67, 2.72
Gestational		1.44	0.66, 3.17
None		1.00	
Hypertensive disorder[c]	36		
Any		1.25	0.92, 1.69
Pre-eclampsia		1.20	0.74, 1.94
Gestational hypertension		1.28	0.88, 1.86
None		1.00	
Participant Factors:			
Maternal age at birth/birth order	9		
<20 years, 1st		1.38	1.04, 1.83
≥20 years, 1st		0.83	0.60, 1.15
<20 years, 2nd or later		1.04	0.70, 1.55
≥20 years, 2nd or later		1.00	
Birth weight, grams	42		
<2,500		1.32	1.03, 1.69
2,500-3,999		1.00	
≥4,000		0.90	0.55, 1.47
Gestational age at birth	68		
Born early, ≥1 months		1.54	1.06, 2.24
Born early, 2-4 weeks		1.10	0.63, 1.91
Not ≥2 weeks early		1.00	
Multiple birth	7		
Any		1.39	1.00, 1.93
Monozygotic		1.89	1.27, 2.80
Dizygotic		0.95	0.54, 1.69
None		1.00	

Supplemental Material, Table 3 (cont.)

Exposure	Imputed %	Imputed (n = 3,534)[b] RR	95% CI
Fed breast milk[c]	17		
Definitely/probably		1.19	1.00, 1.41
Definitely not/probably not		1.00	
Fed soy formula[c]	24		
Definitely/probably		1.30	0.82, 2.06
Definitely not/probably not		1.00	

Abbreviations: risk ratio, RR; CI, confidence interval; DES, diethylstilbestrol.

[a] Early onset uterine fibroid status was based on self-reported diagnosis at ≤ 30 years of age. Multiple imputation by chained equations (MICE) was performed using IVEware Version 0.1 with n = 10 imputations and n = 10 iterations. All early-life factors shown in this table, maternal vital status at baseline, early onset fibroid status, childhood family income, and participant's age and highest level of education were included in the multiple imputation regression model.

[b] Each log-binomial regression model included the following core variables: participant's age and education and maternal age/birth order index. Log-binomial regression estimates for each early-life factor were combined for the 10 imputation datasets using PROC MIANALYZE in SAS Version 9.2.

[c] Definitely and probably were combined as affirmative responses, and probably not and definitely not were combined as negative responses.
